# Early recurrent feedback facilitates visual object recognition under challenging conditions

**DOI:** 10.3389/fpsyg.2014.00674

**Published:** 2014-07-01

**Authors:** Dean Wyatte, David J. Jilk, Randall C. O'Reilly

**Affiliations:** ^1^Department of Psychology and Neuroscience, University of Colorado BoulderBoulder, CO, USA; ^2^eCortex, Inc.Boulder, CO, USA

**Keywords:** object recognition, feedback, top–down attention, illusory contours, amodal completion

## Abstract

Standard models of the visual object recognition pathway hold that a largely feedforward process from the retina through inferotemporal cortex leads to object identification. A subsequent feedback process originating in frontoparietal areas through reciprocal connections to striate cortex provides attentional support to salient or behaviorally-relevant features. Here, we review mounting evidence that feedback signals also originate within extrastriate regions and begin during the initial feedforward process. This feedback process is temporally dissociable from attention and provides important functions such as grouping, associational reinforcement, and filling-in of features. Local feedback signals operating concurrently with feedforward processing are important for object identification in noisy real-world situations, particularly when objects are partially occluded, unclear, or otherwise ambiguous. Altogether, the dissociation of early and late feedback processes presented here expands on current models of object identification, and suggests a dual role for descending feedback projections.

## Introduction

Visual object recognition has traditionally been described as a largely feedforward process that operates independently of and prior to top–down signals that reflect strategic processing or attentional effects. This standard model of object recognition is supported by research that spans multiple levels of analysis, including single- and multi-unit recording, computational modeling, and behavioral experiments, all of which have been discussed in detail in recent reviews (e.g., Serre et al., 2007; DiCarlo et al., 2012). Feedback projections, nearly equal in density to feedforward neurons throughout the ventral visual stream (Felleman and Van Essen, 1991; Sporns and Zwi, 2004), are commonly thought to subserve slower, attention-mediated processing that happens after recognition processes are complete, but not the core object recognition processing itself (Hochstein and Ahissar, 2002).

The proposal advanced in this paper is that these local, recurrent feedback connections also provide an avenue for rapid top–down signals that influence object recognition-related processing *as it is being carried out*—well before the slower attention-mediated processes. The theory is inspired by the pioneering work of Dehaene et al. (2006) and Lamme (2003, 2006) in identifying the neural correlates of consciousness. Both of these researchers' theories dissociate between local recurrent processing and top–down signals from frontoparietal areas in terms of the effects that they have on awareness. The present work draws a similar distinction between top–down, attention-mediated processing, and local recurrent processing between hierarchically adjacent areas within the ventral stream.

We support this distinction by first providing evidence that there are two temporally dissociable processes operating on these feedback projections; and second by presenting results showing an important functional role for the earlier, local recurrent processing.

## Evidence for a temporal dissociation of local recurrent and top–down processing

Top–down attention is known to be a consciously generated, executive signal originating in frontal and parietal areas (Thompson et al., 2005; Bressler et al., 2008). Signals reflecting these strategic processes do not manifest in early visual areas until 150–170 ms after stimulus onset at the earliest, with most reported effects occurring within the range of 200–300 ms (Mehta et al., 2000a,b; Martinez et al., 2001; Noesselt et al., 2002). The relatively long latency of attentional effects in early visual areas is thought to arise from top–down signals that target late stages of the ventral stream and then progress backward toward V1 (Buffalo et al., 2010). Local recurrent processing can also be thought of as a top–down process, except that the signal originates from within the ventral stream itself, as opposed to frontal or parietal areas. Local recurrent processing is completely involuntary and does not require conscious execution, evidenced by its observation in recordings from anesthetized animals (Roland et al., 2006; Roland, 2010) and is simply a consequence of signal propagation through recurrent corticocortical connectivity. Specifically, as soon as a given area responds, signals are routed both to higher-level and lower-level connected areas. Feedback to immediately lower levels occurs with very short latencies—as quickly as 10 ms after the initial feedforward responses (Hupé et al., 2001; Pascual-Leone and Walsh, 2001)—and thus could plausibly be underway after initial feedforward IT neural responses (ca. 80–100 ms) but before the completion of the categorization process (ca. 150 ms).

Recent research using methods that temporarily interfere with cortical processing have revealed strong evidence that recurrent feedback circuits are engaged during the first 80–150 ms of visual processing. One line of evidence comes from experiments that use transcranial magnetic stimulation (TMS) to temporarily prevent a targeted brain area from responding. In a recent study, Koivisto et al. (2011) used fMRI-localized TMS to selectively inactivate V1/V2 while subjects categorized images according to whether they contained an animal. The authors found that applying TMS to V1/V2 with stimulus onset asynchronies (SOAs) of 90–210 ms impaired categorization performance and subjective perception of stimuli. Camprodon et al. (2010) observed similar results with TMS applied over V1 only, but found that there were actually two windows of impairment with SOAs of 100 and 220 ms. Earlier work from Corthout et al. also found an early window of activity with an SOA of around 100 ms during which applying TMS over V1 impairs letter recognition (Corthout et al., 1999a,b). Collectively, these experiments show that disruption of processing in early visual areas around 100 ms after stimulus presentation impairs visual recognition. Importantly, this time window occurs after the earliest contributions of IT neurons, opening up the possibility that the impairment is due to the disruption of feedback to lower-level areas in influencing the quality of object representations. Furthermore, several of these studies found a second, later time window around 200 ms during which TMS also impaired recognition. This later time window coincides with the latency of spatial attention-mediated processing (Mehta et al., 2000a,b; Martinez et al., 2001; Noesselt et al., 2002; Buffalo et al., 2010), providing a temporal dissociation from the rapid recurrent processing effects that are of interest here.

Visual backward masking experiments have also identified a similar time window for recurrent processing around 100 ms after stimulus onset (Fahrenfort et al., 2007, 2008; Boehler et al., 2008). In backward masking experiments, a first stimulus (the target) is followed by a second stimulus (the mask) at a particular latency. At very short latencies, backward masking can impair recognition of the target and in some cases, prevent it from reaching awareness (Macknik and Livingstone, 1998). While the effect of backward masking was initially accounted for with a feedforward explanation (Breitmeyer and Ganz, 1976), modern theories of backward masking emphasize recurrent processing between higher-level and lower-level areas (Enns and Di Lollo, 2000; Lamme and Roelfsema, 2000; Wyatte et al., 2012a). Specifically, if information about a target stimulus being processed in higher-level areas is fed back down to lower areas, but a masking stimulus is simultaneously being processed at that lower level, there will be a fundamental mismatch in the information being processed at each level (Lamme and Roelfsema, 2000). This mismatch causes a decoupling in the functional connectivity (i.e., co-activation) between the visual areas involved in processing the stimulus, which has the psychological effect of greatly reduced perceptual visibility (Dehaene et al., 2001; Haynes et al., 2005).

Boehler et al. (2008) combined a backward masking paradigm with magnetoencephalography (MEG) recording to determine the time course of recurrent feedback to V1 during a recognition task. On trials where subjects correctly recognized the target stimulus (i.e., no impairment from the mask), there was modulation of the V1 MEG signal from 100 to 120 ms. This modulation occurred soon after (ca. 27 ms) the initial V1 signals and almost immediately after (ca. 11 ms) extrastriate generated signals, in strong accordance with being driven by rapid recurrent feedback from extrastriate areas to V1. Again, these rapid recurrent processing effects were dissociable from slower attentional modulation, which manifested 250–300 ms after stimulus presentation and only when subjects attended to the same region of the display that the target appeared in. In contrast, modulation from rapid recurrent processing occurred regardless of where subjects directed attention. Similar results have been demonstrated when combining backward masking with electroencephalography (EEG) recording with both rapid recurrent and slower attentional modulation, but with less emphasis on the specific neural generators of effects given the relatively poor spatial resolution of EEG (Fahrenfort et al., 2007, 2008).

Together, TMS and backward masking experiments provide strong support for the idea that recurrent visual processing engages striate and extrastriate areas around 100 ms after stimulus onset during visual recognition tasks. This local rapid recurrent processing is dissociable from attention-mediated or strategic processing both in terms of where the signals originate (within the ventral stream vs. frontal and parietal areas) and in terms of their relative time courses (ca. 100 ms vs. 150–170 ms at the earliest). Attention has long been known to modulate early EEG responses such as the P1 (first positive deflection, ca. 100 ms) and N1 (first negative deflection, ca. 150–200 ms) (Luck et al., 1990a; Hillyard and Anllo-Vento, 1998). Given the data reviewed here, it seems plausible that the P1 indexes recurrent feedback generated within the ventral stream while the N1 reflects the first influences of frontal and parietal attentional signals that progress backwards through visual areas toward V1 (Buffalo et al., 2010; see also Luck et al., 1990b).

While TMS impairment around 100 ms is consistent with the disruption of recurrent processing, it cannot rule out the possibility that the TMS is actually disrupting delayed feedforward responses. Specifically, low-level image properties such as local contrast can affect the temporal order of feedforward spikes, with lower contrast image regions exhibiting delay relative to more salient image regions (VanRullen and Thorpe, 2001, 2002). However, the information content of these regions is much lower than the salient regions that exhibit fast responses and thus it is unlikely that disrupting their contribution to object recognition processing will negatively impact recognition ability for relatively unambiguous images. More importantly, backward masking experiments that target impairment of recurrent processing provide additional constraints in interpreting TMS effects. Finally, 90–110 ms post stimulus onset is hypothesized to be the time at which peak feedback signals arrive at V1 from extrastriate areas (Roland et al., 2006; Roland, 2010).

**Figure 1:**
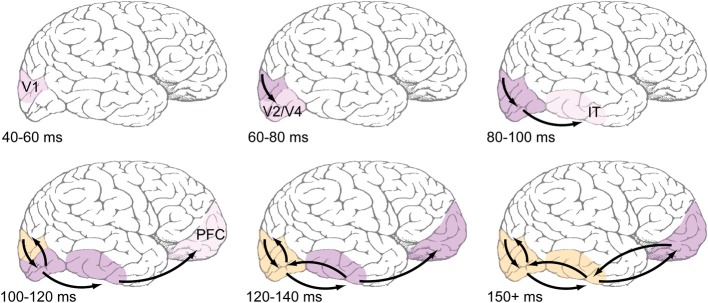
**Proposed time course of feedforward and feedback events during early visual processing. Top row**: Feedforward-dominant latencies, which are well-documented in the literature (e.g., Nowak and Bullier, 1997). Light pink shading refers to earliest reported latencies, likely corresponding to the depicted areas' first spikes, while darker pink shading corresponds ongoing feedforward responses. **Bottom row:** Areas are shaded orange when they are known to be receiving recurrent feedback. Most reports of recurrent feedback to V1 center around an absolute latency of 100 ms after stimulus presentation, with some reports being slightly faster. Common methods used to detect feedback (coarse application of TMS, MEG, EEG) do not have the spatial resolution to distinguish between feedback to V1 and extrastriate areas, but the view taken here is that feedback originates in immediately adjacent areas, and thus those areas that fire earliest during the feedforward dominant phase will also be the first to receive feedback.

Overall, it seems clear that recurrent processing operates within the established time course of object recognition, which spans the first 150 ms of visual processing. The data reviewed in this section are summarized in Table 1 with a rough sketch of overall feedforward and feedback events shown in Figure 1. Having established support for the idea that recurrent feedback occurs rapidly beginning around 100 after stimulus presentation, this paper now turns to discussion of its function.

**Table 1:**
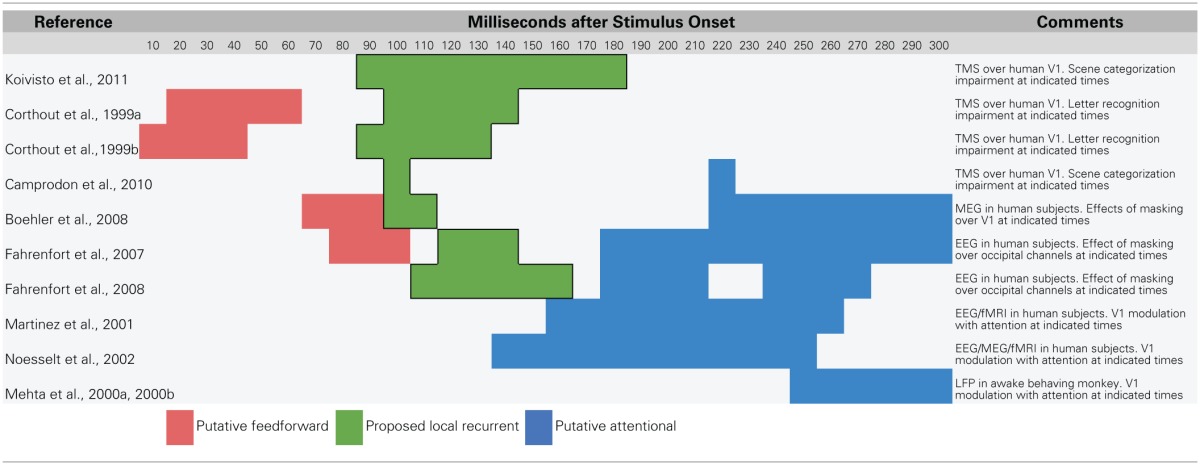
**Summary of data that suggest a temporal dissociation of local recurrent and top–down attentional processing**.

## Evidence for a distinct functional role for local recurrent signals

There is considerable evidence that local recurrent processing is important when stimuli are degraded, partial, or otherwise ambiguous, and we hypothesize that this is one important functional role for the dissociated process described in the previous section. The basic logic behind this proposal is that degrading a stimulus has been shown to weaken the initial responses in object-selective areas (Sclar et al., 1990; Kovacs et al., 1995; Nielsen et al., 2006; Williford and Maunsell, 2006), but recurrent processing over time can strengthen responses back to near undegraded levels and preserve selectivity via top–down reinforcement. Consistent with this idea, object-selective responses in IT cortex remain intact when stimuli are occluded, but take significantly more time to manifest than when stimuli are unoccluded (around an extra 50 ms on average, Kovacs et al., 1995; Nielsen et al., 2006).

Single-unit recordings that use reversible cooling to temporarily inactivate a particular brain area provide further support for our hypothesis. Hupé et al. (1998) applied cooling to area V5/MT, a visual area in the superior temporal sulcus of the monkey brain that sends feedback projections to areas V1, V2, and V3. Recordings from V1 through V3 indicated that responses to moving bar stimuli were vastly weakened (fewer spikes observed per second) when V5/MT was inactive compared to control experiments in which it was active. This attenuation of lower-level responses was most dramatic in low salience conditions, such as when the bar had a very low contrast, a point that will be discussed in further detail later in this section. These results suggest that when higher-level visual areas are active, they provide additional excitatory input to lower levels. Similar effects have been shown for other recurrent circuits in other mammalian species such as those involving middle suprasylvian (MS) cortex and V1 (Galuske et al., 2002) as well as V2 and V1 (Sandell and Schiller, 1982; Mignard and Malpeli, 1991), suggesting that top–down amplification is a highly generic mechanism that occurs between any two recurrently connected areas.

Top–down amplification promotes visual awareness (Lamme, 2003, 2006; Dehaene et al., 2006), and some data indicate that amplification is a simple contrast gain operation, as some have suggested is implemented by attention (e.g., Reynolds et al., 2000; Reynolds and Heeger, 2009). However, there is mounting evidence that recurrent amplification also plays an important functional role in visual object recognition when stimuli are degraded or ambiguous, by promoting a complex grouping and “filling-in” process.

Wyatte et al. (2012a) degraded visual object stimuli using visual occlusion and contrast degradation and used backward masking to control whether recurrent processing mechanisms were available (Enns and Di Lollo, 2000; Lamme and Roelfsema, 2000). When relatively clear stimuli were masked using a relatively long latency 100 ms SOA pattern mask, there was little impairment in recognition performance. However, when heavily occluded or low contrast stimuli were masked, the mask had a much larger effect, suggesting that recurrent processing was crucial in resolving object identity in these conditions. Simulations using a computational model of object recognition that included recurrent feedback between hierarchically adjacent layers (O'Reilly et al., 2013) showed that responses in both lower layers (corresponding to striate/extrastriate regions) and upper layers (corresponding to IT cortex) strengthened over time when objects were occluded. Backward masking selectively interfered with this strengthening process, which was crucial when stimulus signals were weak due to degradation. Furthermore, the strengthening dynamic was found to be specifically due to recurrent feedback—purely feedforward versions of the model exhibited asymptotic response levels across areas.

**Figure 2:**
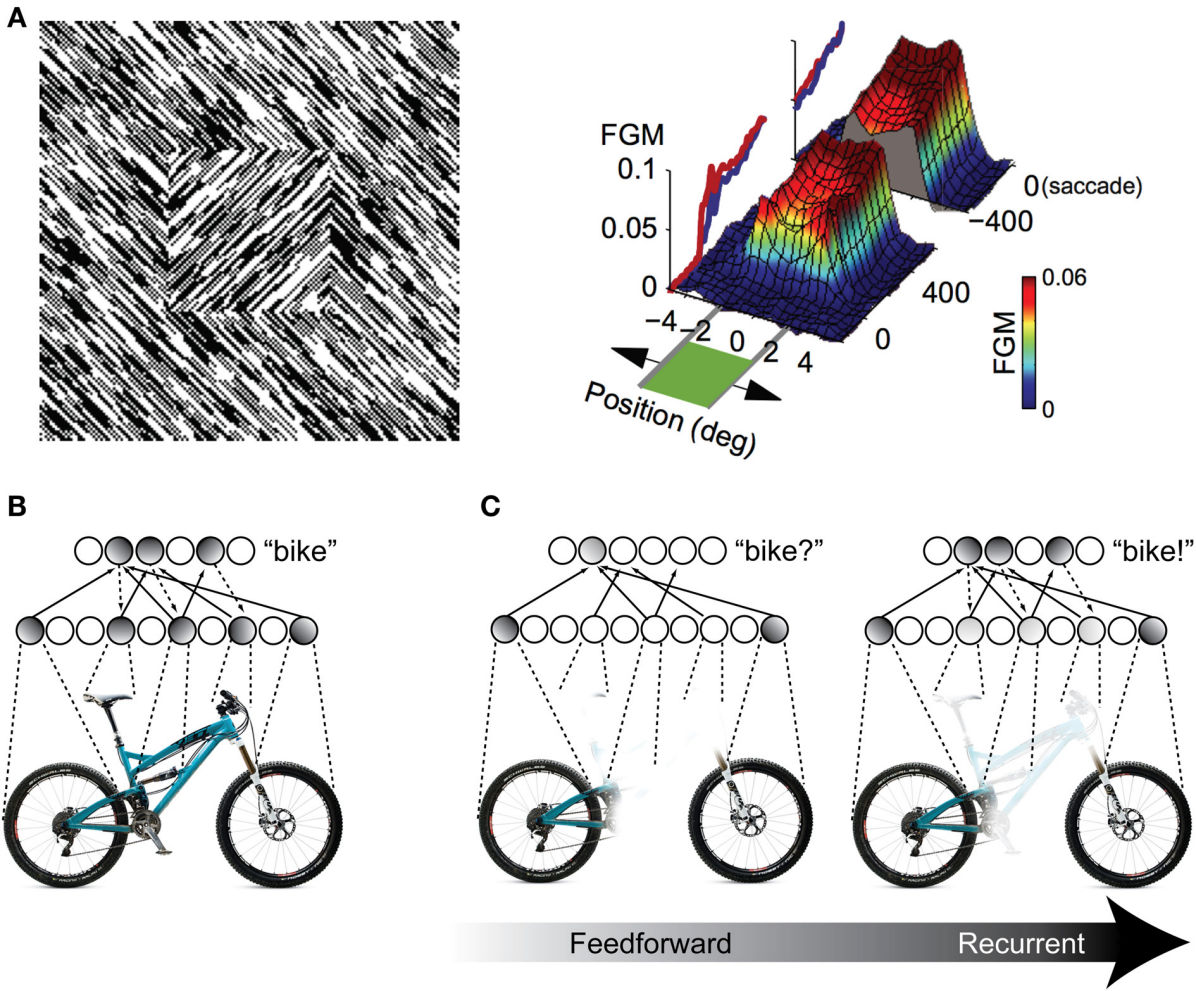
**Illustration of recurrent processing's filling-in computations during figure-ground processing and object recognition. (A)** Processing of an orientation-defined square stimulus results in enhancement of the figural elements compared to the background elements. This enhancement comes in the form of recurrent feedback that groups together common image elements and spreads activation throughout the interior of the square, effectively “filling” it in as a perceptually salient surface. FGM, Figure Ground Modulation, i.e., difference between figure and background responses. Adapted from Lamme et al. (1998) and Poort et al. (2012). **(B,C)** The same feedback-based “filling-in” principle can be applied to object recognition processing when stimuli are occluded. When object features are occluded, only a partial representation is elicited by the first feedforward responses. However, recurrent feedback (e.g., between IT and extrastriate areas) propagates these partial responses back to early visual areas, driving neurons that respond to co-occurring features that might be occluded in the physical stimulus. This recurrent processing between hierarchically adjacent visual areas can effectively “fill in” the occluded features in the object representation.

One possibility for the mechanism underlying these recognition performance differences is a grouping and “filling-in” process similar to what is observed in the figure-ground literature in V1 (Figure 2A), but repeated between higher levels of the visual hierarchy. As an illustration, consider a population of IT neurons that respond to bicycle stimuli (Figures 2B,C). If a bicycle stimulus is occluded and only the wheels are visible, some members of this population will become active (specifically, those corresponding to wheel-like features), but the selective response across the full population will be unavailable. The partial responses, however, will be propagated back to earlier visual areas, which will drive neurons that are sensitive to visual features that are known to co-occur with bicycle wheels, such as a bicycle's frame, handlebars, and saddle. Importantly these responses occur in the absence of these features in the actual stimulus. These “illusory” responses in turn provide new driving potential to IT neurons, ultimately evoking the selective response corresponding to the unoccluded stimulus across the full IT population responsive to bicycles. The IT response is “object complete,” meaning that there is little-to-no difference between the response to the partially occluded object and the complete object—the brain has filled in the missing information.

Computationally, recurrent processing's amplification effect is capable of supporting a grouping or surface-based encoding. The most convincing demonstrations of these computations are found in the figure-ground processing literature, where the term “contextual modulation” is used to describe them (Zipser et al., 1996; Lamme et al., 1998). In contextual modulation, neurons with non-overlapping receptive fields such as those found in V1 are capable of modulating and reinforcing each other by virtue shared connections through higher levels in the visual hierarchy where receptive fields do tend to overlap. This extra modulation has the effect of grouping together figural elements of a display and enhancing their activity relative to background elements effectively spreading activation throughout the figure interior and “filling” it in as a perceptually salient surface (Figure 2A). The models suggest that contextual modulation is driven by recurrent feedback, because lesions of feedback from extrastriate and dorsal structures to V1 obliterate the surface filling effect. They further illustrate that the timing of contextual modulation to area V1 would be on the order of 80–100 ms after stimulus presentation, coinciding with the known time course of feedback to striate areas during visual processing. Finally, contextual modulation is dissociable from slower top–down attentional effects, not just with respect to time course but also because its surface filling computations are retained even when attention is deployed away from the target stimulus (Poort et al., 2012).

**Figure 3:**
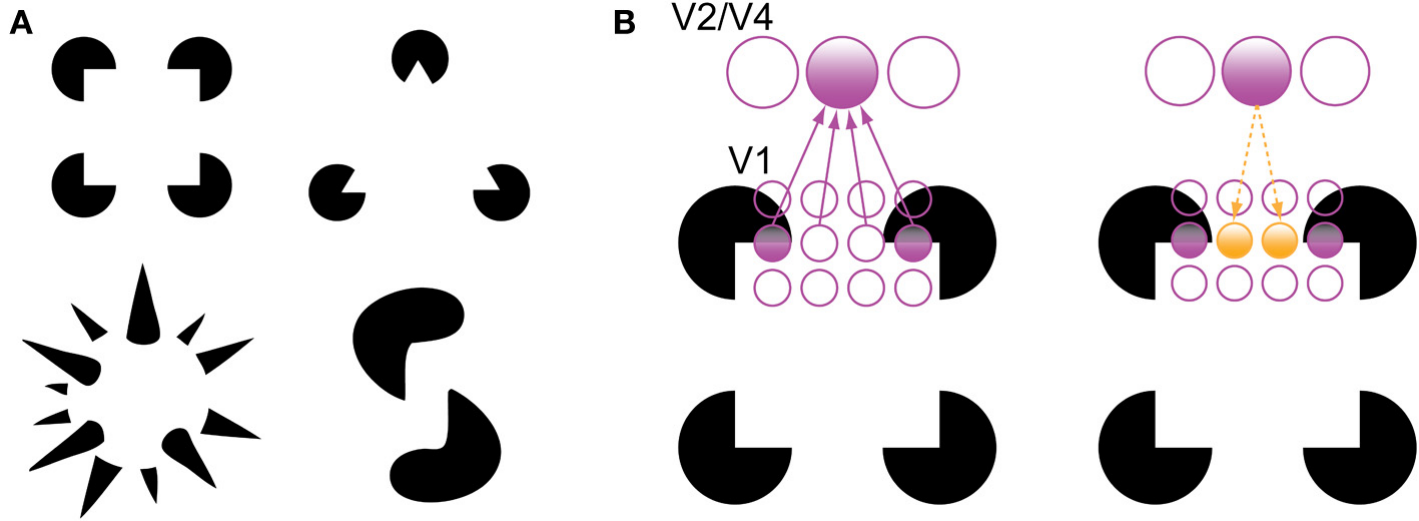
**Illusory contour perception in Kanizsa shapes (Kanizsa, 1979). (A)** Traditional and more complex Kanizsa shapes that evoke strong illusory contour percepts. Complex shapes courtesy of Steven Lehar (http://cns-alumni.bu.edu/~slehar/Lehar.html). **(B**) Perception of illusory contours has been suggested to arise by virtue of recurrent feedback from extrastriate areas to V1. Specifically, feedback from an extrastriate neuron drives neurons that code similarly oriented contours that fall within its receptive field, spreading activation across the gap in the Kanizsa shape. Adapted from Lee (2003).

There are two phenomena in the experimental literature that support the grouping and filling-in roles of recurrent processing during object recognition. The first is the perception of illusory contours, such as in displays containing Kanizsa shapes (Figure 3). V1 neurons have been shown to respond to the illusory contours that compose Kanizsa shapes, such as the edges of the illusory square in Figure 3. Multi-unit recordings have indicated that these responses occur beginning around 100 ms after stimulus presentation, which is shortly after the V1 responses to a physical contour with the same orientation and location, suggesting a role for feedback in their encoding (Lee and Nguyen, 2001; Seghier and Vuilleumier, 2006). Specifically, recurrent feedback from extrastriate areas could support the perception of illusory contours in the Kanizsa illusion by grouping similarly oriented contours at the V1 level that fall within the shape's receptive field; this would cause the shape to be perceived as perceptually salient surface similar to the way texture-defined shapes are perceived (Figure 3B). As such, a recent experiment has indicated that global contour information emerges in V1 responses shortly after the first V4 responses, implicating recurrent feedback in this grouping process (Chen et al., 2014).

The second supportive phenomenon is an actual object completion effect, which has gained support from fMRI studies that show little-to-no difference in the activation levels of occluded and unoccluded stimuli in object-selective regions of cortex (Lerner et al., 2002). Intact activation, however, could simply reflect increased gain of the encoded object fragments without a more complex completion process. To differentiate between these two possibilities, one can use an fMRI adaptation paradigm, which depends on neural mechanisms that decrease response levels for repeated stimuli that are perceived as the same. This method gives an experimenter an index of how perceptually similar two experimental conditions are. For example, Kourtzi and Kanwisher (2001) presented observers with images that contained occluding bars either in front of or behind target objects (in which case, the targets were effectively unoccluded). The experiment measured the hemodynamic response in the lateral occipital cortex (LOC), which has been strongly suggested as the human homolog of IT cortex in monkey (Grill-Spector et al., 2001; Orban et al., 2004). The results indicated that there was no significant change in hemodynamic response when subjects were presented with two identical objects in sequence, as well as when subjects were presented with occluded and unoccluded versions of an object in sequence. Thus, at the level of LOC, there is little difference in the way that unoccluded and occluded versions of the same object are represented. More recent techniques such as representational similarity (Kriegeskorte et al., 2008a,b) or decoding analyses (Tang et al., in press) might further illuminate how occluded objects are represented in various regions of cortex.

While the perception of illusory contours has been linked to recurrent feedback (Lee, 2003), this explanation has not been has explored as extensively in the object completion literature, likely due to most studies using relatively coarse measures like fMRI (e.g., the aforementioned studies that rely on fMRI adaptation). Computationally, illusory contour perception and object completion could be implemented by the same mechanism, whereby higher-level neurons with overlapping receptive fields feed responses back to lower-level neurons in the absence of the visual information itself and produce the perception of illusory object features. According to this view, when operating between extrastriate levels and V1, the mechanism produces illusory contours; when operating between IT cortex and extrastriate areas, it produces more complex illusory object features. There is some support for this idea in the literature. For example, Rauschenberger et al. (2006) demonstrated object completion effects in LOC as well as in extrastriate areas when stimuli were presented for longer durations, suggesting that there is a “temporal unfolding” of object completion from higher levels of the ventral stream to lower-level areas.

However, illusory contour stimuli evoke a perceptually salient completion phenomenon, whereas the filling-in of objects does not. These processes have been distinguished in the literature as “modal” and “amodal” completion, respectively (Johnson and Olshausen, 2005; Seghier and Vuilleumier, 2006). Modal completion has been shown to elicit illusory responses in V1 (Lee and Nguyen, 2001), supporting the idea that whatever representation is present in V1 is what we “perceive” (Bullier, 2001). It is unclear whether amodal completion processing also reaches back to the level of V1. Some studies indicate that V1 represents completed shapes (Rauschenberger et al., 2006), whereas others show that the complete representation is only present in extrastriate and higher-level areas (Weigelt et al., 2007). More recently, Emmanouil and Ro (2014) showed that object completion can occur rapidly and without visual awareness, further supporting the dissociation of object completion from top–down attention.

If our proposal is correct, the time course of object completion effects should agree with the time course of recurrent processing as described above. Some studies show object completion effects beginning to manifest over temporal and parietal sites (as indexed by EEG scalp recordings) around 130 ms at the earliest and continuing to evolve until around 200 ms into processing (Johnson and Olshausen, 2005; Chen et al., 2009). These data are consistent with the explanation of object completion rapidly engaging recurrent processing with striate and extrastriate areas, assuming the 50 ms delay typically observed when the brain is processing occluded object stimuli (Kovacs et al., 1995; Nielsen et al., 2006).

However, other studies have suggested a much later time course for object completion effects, beginning around 200 ms and completing around 400 ms (Doniger et al., 2000; Sehatpour et al., 2006, 2008). One consistent characteristic of these latter studies is that they use fragmented line drawings of objects, whereas studies that associate an early time course with object completion have used photorealistic images of objects. It is unclear whether this late temporal correlate of object completion is due to relatively slow, attention-mediated processing, or due to a fundamentally different type of processing. For example, photorealistic occlusion might recruit the surface-coded computations associated with recurrent processing since there are explicitly depicted depth planes (an occluder and an object) whereas resolving contour fragmentation might rely on a completely different computation since depth planes are less well-defined in line drawings. Furthermore, the studies that associate the later time course with object completion have not used a paradigm such as response adaptation that crucially allows inference about whether an unoccluded and occluded object are represented similarly.

In summary, it seems clear that recurrent processing promotes signal amplification between reciprocally connected brain regions. There is substantial evidence that this is not a simple multiplicative gain operation, but a considerably more complex grouping or surface-based computation that spreads activation between related object features. This idea has been well-studied in the literature on illusory contour perception and the data support the explanation that illusory contour perception is due to V1 neurons receiving recurrent feedback from extrastriate regions. The same idea can be applied to object completion effects in IT cortex, predicting that they are due to feedback-rectified signals from extrastriate regions. This recurrent processing-based explanation has received little attention in the literature, but is generally supported by the timing of object completion effects.

## Summary and future research

Over the last 5–10 years, evidence has accumulated that local recurrent signals are an integral part of early visual processing. TMS studies have indicated that recurrent processing engages striate and extrastriate areas during visual recognition tasks in as little as 100 ms (Camprodon et al., 2010; Koivisto et al., 2011) and theories of backward masking have provided additional accordant timing data as well as suggested a general theory of how corticocortical interactions support visual perception (Fahrenfort et al., 2007, 2008; Boehler et al., 2008). Surprisingly though, relatively little work has focused on synthesizing these ideas with theories of visual object recognition, which is commonly held to be primarily a feedforward process (DiCarlo et al., 2012). Instead, theories of recurrent processing have focused on the role of interactions between brain areas in promoting visual awareness (Lamme, 2003, 2006; Dehaene et al., 2006). Object perception has long been known to benefit from top–down signals that reflect attention or strategic processing, but its time course has been considered to be too slow to support the initial rapid recognition processes (Hochstein and Ahissar, 2002; VanRullen, 2007).

This paper has attempted to map out the time course of feedforward- and feedback-based events during the first 150 ms of visual processing and establish the function that rapid recurrent processing between brain areas plays within this time frame. Specifically, we propose the following overall process: A feedforward-dominant wave of activation flows up to IT in the first 80–100 ms after stimulus presentation, quickly evoking object-selective responses, while, simultaneously, activation is also feeding backward through this pathway. In the following 20 ms (an absolute latency of 100–120 ms), prefrontal areas that support the actual object categorization decision receive their first feedforward responses from IT neurons, while simultaneously, recurrent feedback from extrastriate areas has had sufficient time to more fully engage V1 populations. Recurrent feedback to V1 amplifies neurons' initial responses by grouping the responses to similar object features and enhancing them relative to other responses (Zipser et al., 1996; Lamme et al., 1998; Poort et al., 2012). In some cases, these grouping computations can cause the perception of illusory contours and surfaces (Lee and Nguyen, 2001; Seghier and Vuilleumier, 2006), but they also seem to be important when objects are degraded in order to rectify signals (Hupé et al., 1998). At an absolute latency of 120–140 ms after the initial stimulus presentation, the now extensive recurrent processing between IT and extrastriate areas can cause the representation of more complex illusory features that support object completion, by propagating these illusory responses back toward IT populations. We have recently developed a biologically-based computational model that exhibits just these dynamics (O'Reilly et al., 2013), and can provide a platform for integrating the various data cited here, while generating further testable predictions.

It is unlikely that object completion in IT cortex is a sole function of rectified responses from extrastriate areas being propagated forward in the range of 120–140 ms (or 170–190 ms, assuming the 50 ms delay observed when the brain processes occluded object stimuli; see Kovacs et al., 1995; Nielsen et al., 2006). Object completion likely also benefits from the first recurrent responses from prefrontal areas that arrive shortly after this time frame. This feedback from prefrontal areas could reflect top–down predictions that constrain the space of potential object representations in IT cortex (Bar et al., 2006; Kveraga et al., 2007), which might also have the effect of filling in visual information when it is missing from the physical stimulus. It is also plausible that lateral interactions within IT cortex itself could support object completion by enforcing statistical co-occurrences and mutual exclusions between object features (Akrami et al., 2009; Daelli and Treves, 2010). It would not be surprising if a combination of rectified feedforward responses, feedback from prefrontal areas, and lateral interactions within IT cortex itself support object completion by bringing the brain as a whole into an attractor that combines bottom–up sensory information with top–down task demands and appropriate local constraints (e.g., Spivey, 2008). Future research that uses sophisticated techniques to rapidly and systematically disable feedforward, recurrent and lateral connectivity (e.g., optogenetics, Deisseroth, 2011) might be necessary to disentangle the relative contributions of each of these influences. Nevertheless, any contribution to object completion from local recurrent processes is supportive of the distinct functional role in resolving degraded or ambiguous stimuli proposed here.

One remaining question concerns whether recurrent processing is necessary for recognizing relatively unambiguous stimuli. “Core object recognition” (DiCarlo et al., 2012) of stimuli that vary in terms of their spatial position, scale, pose, and illumination can be rapidly decoded from the first IT responses (Hung et al., 2005). Early IT responses are also known to exhibit invariance to limited clutter (Missal et al., 1997; Zoccolan et al., 2005), suggesting that the bulk of object recognition is solved by a largely feedforward process. Importantly, these data are not fundamentally incompatible with the theory proposed here. Feedback acts on immediately lower areas with latencies as short as 10 ms (Hupé et al., 2001; Pascual-Leone and Walsh, 2001) and might be important for the Winner-Take-All (WTA) or “max” computations (Riesenhuber and Poggio, 1999; Wyatte et al., 2012b; O'Reilly et al., 2013) that have been suggested to contribute to core object recognition. Our theory has focused on recurrent processing under challenging object recognition conditions such as when stimuli are occluded or otherwise degraded. However, more substantial variability in the spatial properties of inputs might also benefit from recurrent processing. A variant of the “animal/no animal” recognition task used in many studies has shown that increasing target viewing distance in the stimulus causes backward masking to have a greater effect (Serre et al., 2007, supporting information), implicating recurrent processing for robust recognition under these conditions (Wyatte et al., 2012a). Further research with stimuli whose spatial properties can be manipulated parametrically (DiCarlo et al., 2012; Cadieu et al., 2013) combined with methods like TMS and backward masking will be necessary to determine the exact conditions under which recurrent processing is necessary.

If the theory proposed here is true, the standard description of object recognition as a feedforward process is somewhat misleading. Simply put, there is always ongoing brain activity that must be combined with new incoming sensory information, so that the notion of a strictly “feedforward sweep” is fundamentally ill-conceived (Arieli et al., 1996; Tsodyks et al., 1999). Ongoing activity could be used to establish moment-to-moment constraints that effectively guide coherent perception via recurrent processing mechanisms. While the seminal research on object recognition often focused on simple spike counts of anesthetized animals to map out the receptive field characteristics of neurons throughout the ventral stream in a well-controlled manner, future research should emphasize more complex corticocortical interactions in the awake, behaving brain to determine how neural interactions involving feedforward, lateral, and recurrent processing mechanisms combine to give rise to the visual system's robust perceptual abilities even in difficult stimulus conditions.

### Conflict of interest statement

The authors declare that the research was conducted in the absence of any commercial or financial relationships that could be construed as a potential conflict of interest.
